# 1216. Presence of the Narrow-Spectrum OXA-1 Beta-lactamase Enzyme Is Associated with Elevated Piperacillin-Tazobactam MIC Values Among ESBL-producing *Escherichia coli* Clinical Isolates (CANWARD, 2007-2018)

**DOI:** 10.1093/ofid/ofab466.1408

**Published:** 2021-12-04

**Authors:** Andrew Walkty, James Karlowsky, Philippe Lagace-Wiens, Alyssa Golden, Melanie Baxter, Andrew Denisuik, Melissa McCracken, Michael Mulvey, Heather Adam, George Zhanel

**Affiliations:** 1 Shared Health, Winnipeg, Manitoba, Canada; 2 University of Manitoba, Winnipeg, Manitoba, Canada; 3 Public Health Agency of Canada, Winnipeg, Manitoba, Canada; 4 National Microbiology Laboratory, Public Health Agency of Canada, Winnipeg, MB, Canada

## Abstract

**Background:**

The clinical outcome of patients with bacteremia due to an extended-spectrum beta-lactamase (ESBL)-producing member of the family *Enterobacteriaceae* who are treated with piperacillin-tazobactam appears to depend, at least in part, on the piperacillin-tazobactam MIC. The purpose of this study was to determine whether there is any association between the MIC of piperacillin-tazobactam and presence of the narrow spectrum OXA-1 beta-lactamase enzyme among ESBL-producing *Escherichia coli*.

**Methods:**

*E. coli* clinical isolates were obtained from patients evaluated at hospitals across Canada (January 2007 to December 2018) as part of an ongoing national surveillance study (CANWARD). ESBL production was confirmed using the Clinical and Laboratory Standards Institute phenotypic method. Susceptibility testing was carried out using custom broth microdilution panels, and all isolates underwent whole genome sequencing for beta-lactamase gene detection.

**Results:**

In total, 671 ESBL-producing *E. coli* were identified as part of the CANWARD study. The majority of isolates (92.0%; 617/671) harbored a CTX-M ESBL enzyme. CTX-M-15 (62.3%; 418/671), CTX-M-27 (13.9%; 93/671), and CTX-M-14 (13.4%; 90/671) were the most common variants identified. The narrow spectrum OXA-1 beta-lactamase enzyme was present in 42.6% (286/671) of isolates. OXA-1 was detected in 66.3% (277/418) of isolates with a CTX-M-15 ESBL enzyme versus only 3.6% (9/253) of isolates with other ESBL enzyme types. The piperacillin-tazobactam MIC_50_ and MIC_90_ values were 8 µg/mL and 32 µg/mL for isolates that possessed the OXA-1 enzyme versus 2 μg/mL and 8 µg/mL for those that did not. The percentage of ESBL-producing *E. coli* isolates that were inhibited by a piperacillin-tazobactam MIC of ≤8 μg/mL was 68.5% for isolates that were OXA-1 positive and 93.8% for isolates that were OXA-1 negative.

**Conclusion:**

The MIC_50_ and MIC_90_ values of piperacillin-tazobactam among ESBL-producing *E. coli* were higher for the subset of isolates that harbored a narrow spectrum OXA-1 beta-lactamase enzyme relative to the subset that did not. This association was primarily observed among ESBL-producers with the CTX-M-15 enzyme variant. OXA-1 was infrequently detected among isolates with other ESBL enzyme types.

**Disclosures:**

**George Zhanel, PhD**, **AVIR** (Grant/Research Support)**Iterum** (Grant/Research Support)**Merck** (Grant/Research Support)**Pfizer** (Grant/Research Support)**Sandoz** (Grant/Research Support)**Sunovion** (Grant/Research Support)**Venatorx** (Grant/Research Support)**Verity** (Grant/Research Support)

